# Phenotype, effector function, and tissue localization of PD-1-expressing human follicular helper T cell subsets

**DOI:** 10.1186/1471-2172-12-53

**Published:** 2011-09-13

**Authors:** Chuanwu Wang, Peter Hillsamer, Chang H Kim

**Affiliations:** 1 Laboratory of Immunology and Hematopoiesis, Department of Comparative Pathobiology; Center for Cancer Research; Bindley Bioscience Center; Purdue University, West Lafayette, IN 47907, USA; 2 Sagamore Surgical Center, Lafayette, IN 47909, USA

## Abstract

**Background:**

It is well established that PD-1 is expressed by follicular T cells but its function in regulation of human T helper cells has been unclear. We investigated the expression modality and function of PD-1 expressed by human T cells specialized in helping B cells.

**Results:**

We found that PD-1-expressing T cells are heterogeneous in PD-1 expression. We identified three different PD-1-expressing memory T cell subsets (i.e. PD-1^low (+)^, PD-1^medium (++)^, and PD-1^high (+++) ^cells). PD-1^+++ ^T cells expressed CXCR5 and CXCR4 and were localized in the rim of germinal centers. PD-1^+ ^or PD-1^++ ^cells expressed CCR7 and were present mainly in the T cell area or other parts of the B cell follicles. Utilizing a novel antigen density-dependent magnetic sorting (ADD-MS) method, we isolated the three T cell subsets for functional characterization. The germinal center-located PD-1^+++ ^T cells were most efficient in helping B cells and in producing IL-21 and CXCL13. Other PD-1-expressing T cells, enriched with Th1 and Th17 cells, were less efficient than PD-1^+++ ^T cells in these capacities. PD-1^+++ ^T cells highly expressed Ki-67 and therefore appear active in cell activation and proliferation in vivo. IL-2 is a cytokine important for proliferation and survival of the PD-1^+++ ^T cells. In contrast, IL-21, while a major effector cytokine produced by the PD-1-expressing T helper cells, had no function in generation, survival, or proliferation of the PD-1-expressing helper T cells at least in vitro. PD-1 triggering has a suppressive effect on the proliferation and B cell-helping function of PD-1^+++ ^germinal center T cells.

**Conclusion:**

Our results revealed the phenotype and effector function of PD-1-expressing T helper cell subsets and indicate that PD-1 restrains the B cell-helping function of germinal center-localized T cells to prevent excessive antibody response.

## Background

Programmed death-1 (PD-1 or also called CD279) is a member of the CD28 family costimulatory molecules [1,2]. Unlike CD28, PD-1 has two intracellular tyrosine signaling motifs (immunoreceptor tyrosine inhibition motif and immunoreceptor tyrosine-based switch motif) [3] and recruits intracellular phosphatase SHP2 (SRC homology 2 domain-containing protein tyrosine phosphatase 2) that dephosphorylates and deactivates downstream signal transducers [4,5]. PD-1 is expressed by a number of immune cell types including activated T cells, B cells, dendritic cells, monocytes, and mast cells in mice. As the ligands for PD-1, PD-L1 (CD274/B7-H1) and PD-L2 (CD273/B7-DC) have been identified [6,7].

In general, engagement of PD-1 by PD-L1 or PD-L2 inhibits TCR-mediated T cell proliferation and cytokine production [8,9], indicating that the cross-linking of PD-1 by its ligands leads to down-regulation of T cell responses in a manner somewhat similar to the effect of CTLA4 stimulation. PD-1-deficient mice are prone to develop autoimmune diseases such as autoantibody formation, dilated cardiomyopathy, acute type I diabetes, and bilateral hydronephrosis [10,11]. In humans, single nucleotide polymorphisms in the PD-1 gene are linked to a number of autoimmune diseases including lupus, rheumatoid arthritis, Graves' disease, type I diabetes, multiple sclerosis, ankylosing spondylitis, and myocardial infarction [12-18]. In mice, blocking of PD-1 exacerbated a lupus-like nephritis [19]. Also, triggering of PD-1 suppressed rheumatoid arthritic symptoms [20]. While PD-1 and its ligands are thought to function to promote immune tolerance, it was also reported that mice deficient in PD and their ligands had fewer long-lived plasma cells, suggesting a certain positive role of PD-1 in regulation of humoral immunity in mice [21].

PD-1 is highly expressed by a subset of T cells in the germinal centers (GC) [22-25]. In contrast, most human B cells do not express PD-1 [22]. Additionally, PD-1 is preferentially expressed on exhausted CD8^+ ^T cells during chronic viral infection [26-29]. Although the suppressive function of PD-1 on CD8^+ ^T cells has been studied extensively, the phenotype and role of PD-1-expressing CD4^+ ^T helper cells in regulation of humoral immune responses have been unclear. We investigated the phenotype and function of PD-1-expressing T helper cells in human tonsils and the function of PD-1 in regulation of these T cells. Our study revealed that PD-1-expressing human helper T cells are heterogeneous in PD-1 expression, chemotactic response, tissue localization, cytokine response, and effector function. Moreover, triggering of PD-1 can restrain the B cell-helping function of the PD-1^high (+++) ^T cells.

## Results

### PD-1-expressing T helper cells are heterogeneous in PD-1 expression and tissue localization in human tonsils

**Figure 1:**
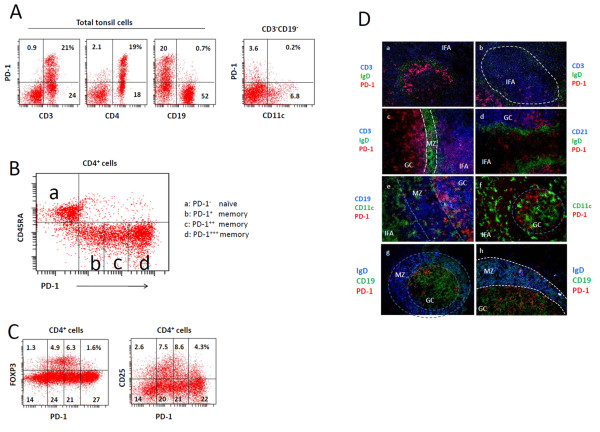
**PD-1-expressing T cells and their localization in human tonsils**. (A) T helper cells, but not DCs and B cells, express PD-1 at high levels in tonsils. (B) Definitions of the PD-1 expressing CD4^+ ^T cell subsets (PD-1^+^, PD-1^++^, and PD-1^+++ ^cells) in this study. (C) Expression of FOXP3 or CD25 versus PD-1 by total tonsil CD4^+ ^T cells. (D) In situ immunofluorescence identification of PD-1-expressing T cells. Frozen tonsil sections were stained with indicated antibodies. A representative data set of at least 3 independent experiments is shown. Interfollicular area (IFA), mantle zone (MZ) and germinal centers (GC) are indicated.

We examined the PD-1 expression by T cells, B cells and dendritic cells in human tonsils. PD-1 was mainly expressed by CD4^+ ^T cells but neither by CD19^+ ^B cells nor CD11c^+ ^dendritic cells (Figure 1A). Among the CD4^+ ^T cells, naïve CD45RA^+ ^T cells were PD-1^-^. However, almost all memory (CD45RA^-^) T cells expressed PD-1 at various levels (Figure 1B). They can be fractionated into three subsets (PD-1^+^, PD-1^++^, and PD-1^+++^) based on the level of PD-1 expression. 15-20% of PD-1 ^dim (+/++) ^cells were FOXP3^+ ^or CD25^+ ^T cells (Figure 1C).

We investigated the localization of the PD-1-expresing T cells. The PD-1^+++ ^cells that expressed PD-1 at the highest level were localized in the outer rim of GC adjacent to the mantle zone (Figure 1D-a). In contrast, PD-1 ^dim (+/++) ^cells were frequently found in either the center of GC or interfollicular areas (IFA; Figure 1D-a and 1b). PD-1^+++ ^T cells were localized close to IgD^+ ^B cells in the mantle zone (Figure 1D-c and 1d). Some PD-1-expressing T cells were found even inside the mantle zone (Figure 1D-e). There was no clear association of the sites of PD-1-expressing T cells and dendritic cells (Figure 1D-f). Both small and large germinal centers had PD-1-expressing T cells (Figure 1D-g and 1h). The numbers and location of these T cells in various primary and secondary follicles demonstrate that PD-1 ^high (+++) ^T cells are the germinal center T cells, while PD-1 ^dim (+/++) ^T cells are in the T cell area or mantle zone.

### PD-1-expressing T cells differentially express secondary lymphoid tissue-homing chemokine receptors

**Figure 2:**
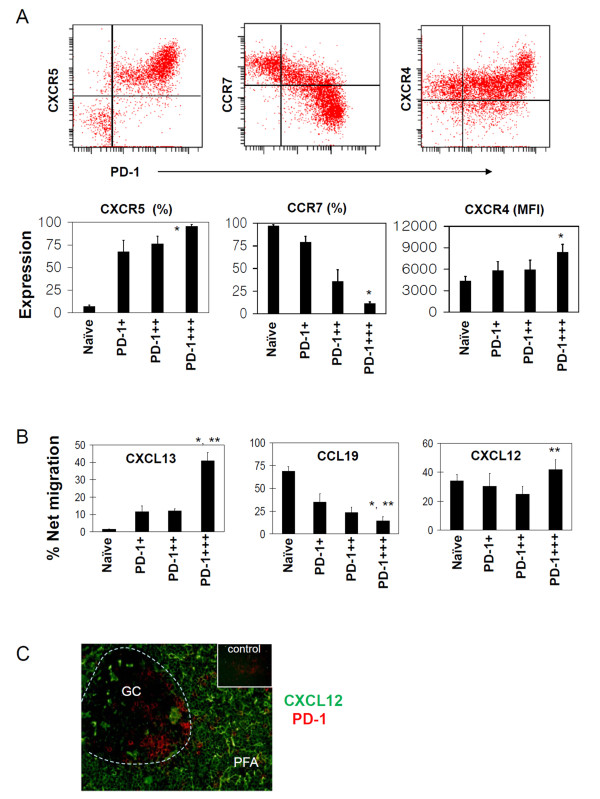
**Expression of chemokine receptors and chemotaxis of PD-1-expressing T cells**. (A) Expression of CXCR5, CCR7 and CXCR4. (B) Chemotaxis to respective chemokine ligands, CXCL13, CCL19 and CXCL12. (C) Expression of CXCL12 in the tonsil follicular area. % Cells expressing indicated chemokine receptors among each group of cells are shown in panel A. %Net migration in panel B indicates specific cell migration levels after subtraction of the background migration occurring in the control medium. The inset in panel C is the image obtained with an anti-PD-1 antibody and an isotype control antibody. Combined (A) or representative data (A, B, and C) of at least 3 independent experiments are shown. "*" and "**"indicate significant differences from naïve T cells and PD-1^++ ^cells respectively. "MFI" stands for mean fluorescence intensity.

The differential localization of PD-1-expressing T cells in human tonsils is intriguing. We investigated the expression of key chemokine receptors, CXCR5 (the CXCL13 receptor), CCR7 (receptor for CCL19 and CCL21), and CXCR4 (the CXCL12 receptor), which are known to regulate the localization of the T cells in secondary lymphoid tissues (Figure 2A). All PD-1-expressing T cells expressed CXCR5, thus meeting the definition of follicular T cells. Compared to PD-1^+/++ ^T cells, however, PD-1^+++ ^T cells even more highly expressed CXCR5. Naïve PD-1^- ^T cells did not express CXCR5. Interestingly, CCR7 expression was exactly the opposite of the CXCR5 expression pattern with PD-1^+++ ^T cells expressing CCR7 at the lowest level. While all of the subsets expressed CXCR4, it was the PD-1^+++ ^T cells that expressed CXCR4 at the highest level.

Next, we examined the activity of the chemokine receptors expressed by the PD-1-expressing T cells (Figure 2B). PD-1^+++ ^T cells were highly responsive to CXCL13 but poorly migrated to CCL19. PD-1^+/++ ^T cells were responsive to both CXCL13 and CCL19 at moderate levels. PD-1^+++ ^T cells were more responsive to CXCL12 than PD-1^++ ^T cells. Overall the chemotactic responses were in line with the expression levels of chemokine receptors.

Because the high expression of CXCR4 by PD-1^+++ ^T cells and their localization in the rim of GC, we examined the in situ expression of CXCL12 at protein level (Figure 2C). It was found that CXCL12 was expressed by the stromal cells in the mantle zone and throughout the interfollicular area but not significantly within GC. It was notable that the site of CXCL12-expression in the mantle zone was found right next to the sites of PD-1^+++ ^T cell localization (Figure 2C). Thus, it appears that PD-1^+++ ^T cells localize to the outer rim of GC adjacent to the mantle zone because of the combined chemotactic force of CXCL12 and CXCL13 in the absence of the chemotactic influence of CCL19/CCL21.

### PD-1-expressing T cells have distinct surface antigen and cytokine production phenotypes

**Figure 3:**
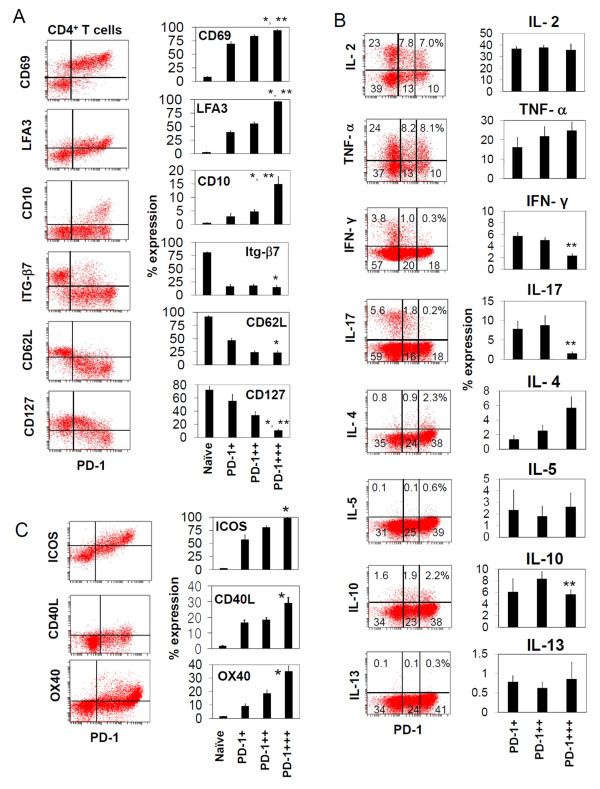
**Expression of surface antigens, cytokines, and costimulatory molecules by PD-1-expressing T cells**. (A) Flow cytometric analysis of the surface antigen expression by freshly isolated tonsil T cells is shown as graphs and dot plots. (B) Intracellular cytokine expression by CXCR5^+ ^PD-1^+ ^CD4^+ ^T cells. The T cells were surface-stained and, then, activated with PMA and ionomycin in the presence of monensin for 4 h. The CXCR5^+ ^PD-1^+ ^CD4^+ ^T cells are gated excluding the largely PD-1-negative naive T cells. Representative data out of 3 independent experiments are shown. (C) Surface expression of ICOS and OX40 and intracellular expression of CD40L by freshly prepared PD-1-expressing T cells. Combined data of 3 independent experiments are shown in the graphs. "*" and "**"indicate significant differences from naïve T cells and PD-1^++ ^cells respectively.

In order to gain more insights into the phenotype of the PD-1-expressing T cells, we examined the expression of various surface antigens such as CD69, CD10, CD127, CD62L, and leukocyte function antigen (LFA)3 (Figure 3A). All PD-1-expressing T cells were CD69^+^. PD-1^+++ ^T cells highly expressed the adhesion molecule LFA3 but not CD62L and integrin β7. CD10 (an angioimmunoblastic T-cell lymphoma marker) was specifically expressed by a subset of PD-1^+++ ^T cells. CD127 (a component of the IL-7 receptor) was decreased on PD-1^+++ ^T cells. Thus, PD-1^+++ ^T cells have the surface phenotype, CD69^+ ^LFA3^+ ^CD10^+/- ^CD127^- ^CD62L^-^. Loss of CD127 is commonly cited as a phenotype specific for FoxP3^+ ^T regulatory cells but the phenotype of PD-1^+++ ^T cells suggests that it is actually a shared phenotype among certain effector or regulatory T cells.

We further investigated the cytokine production capacity and co-stimulation receptor expression property of the PD-1-expressing T cells. All of the three PD-1-expressing T cell subsets were able to produce IL-2 and TNF-α (Figure 3B). PD-1^- ^T cells were largely of naïve T cells and included some cells capable of producing IL-2 (~12%) and TNFα (7%). However, the PD-1-expressing T cells were heterogeneous in production of IFN-γ and IL-17: PD-1^+++ ^cells included few Th1 or Th17 cells, while PD-1^+ ^T cells contained most Th1 and Th17 cells, suggesting that most polarized effector T cells belong to the PD-1^+ ^T group (Figure 3B). PD-1^+++ ^T cells contain a small number (~5%) of IL-4 or IL-10-producing T cells. IL-5 or IL-13 producers were hardly detected. PD-1^+++ ^T cells highly expressed co-stimulator receptors such as OX40 and ICOS (Figure 3C). Also, intracellular CD40L was expressed more highly by PD-1^+++ ^T cells than PD-1^+ ^or PD-1^++ ^cells.

### The PD-1-expressing T cells are heterogeneous in production of CXCL13, cell proliferation and survival

**Figure 4:**
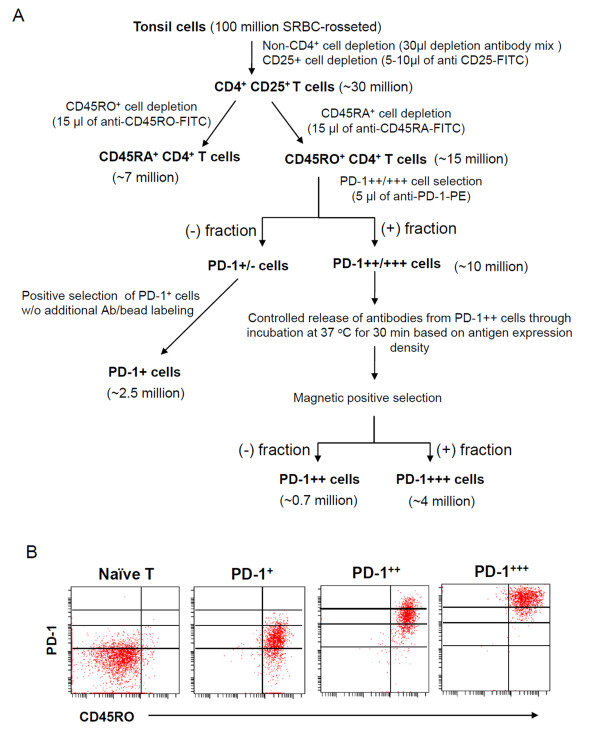
**Isolation of PD-1^+^, PD-1^++^, and PD-1^+++ ^T cells using a novel antigen density-dependent magnetic sorting (ADD-MS)**. (A) ADD-MS procedure utilizing controlled release of antibody/beads based on antigen expression density. The key idea behind this method is to release the bound beads with short term incubation (30 min) at 37°C to differentially sort high and medium antigen-expressing cells. In a typical experiment, approximately (~) 100 million SRBC-rosetted T cells were processed to prepare ~4 million PD-1^+++^, ~0.7 million PD-1^++^, and ~3 million PD-1^+ ^T cells. Anti-FITC or anti-PE microbeads (Miltenyi Biotec) were used at 2× volume of the primary FITC or PE-conjugated antibodies to sort the T cell subsets. (B) PD-1 and CD45RO expression by the CD4^+ ^T cells isolated by the ADD-MS method.

**Figure 5:**
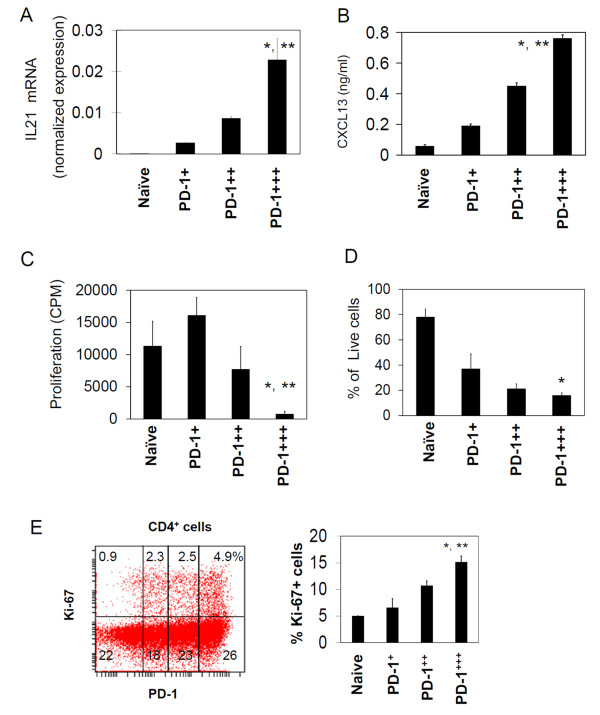
**PD-1-expressing T cells vary in IL21 expression, CXCL13 production, cell proliferation and cell survival abilities**. (A) Expression of IL21 mRNA by the T cell subsets. Real time PCR was performed. Shown are expression levels normalized with that of β-actin. (B) CXCL13 production in response to anti-CD3, anti-CD28, and IL-2. (C) Proliferation responses of PD-1-expressing T cells in response to anti-CD3 and anti-CD28. (D) Cell survival rates after 5 days of culture in complete medium in the absence of TCR activators and cytokines. (E) Expression of Ki-67 by PD-1-expressing T cell subsets. Combined data of 3 independent experiments are shown in the graphs. "*" and "**"indicate significant differences from naïve T cells and PD-1^++ ^cells respectively.

In order to study the function of the PD-1-expressing T cells, we isolated naïve T cells, PD-1^+^, PD-1^++^, and PD-1^+++ ^T cells at high purities (> 97%) utilizing an antigen density-dependent magnetic sorting (ADD-MS) developed for the study (Figure 4). IL-21 is a major cytokine for follicular helper T cells in helping B cells. IL-21 expression by T cells was proportional to their expression levels of PD-1 expression (Figure 5A). We reported previously that a subset of GC-T cells can produce CXCL13 [30]. We determined if the PD-1-expressing T cells have the same phenotype. As shown in Figure 5B, PD-1^+++ ^T cells were most efficient in CXCL13 production. Overall, the CXCL13 production ability of the PD-1-expressing T cells correlated well with the PD-1 expression level. We, next, examined the proliferation and survival ability of the PD-1-expressing T cells. Unlike PD-1^+ ^T cells and PD-1^++ ^cells, PD-1^+++ ^T cells failed to proliferate in vitro in response to stimulation with anti-CD3 and anti-CD28 antibodies (Figure 5C). The PD-1^+++ ^T cells had a poor survival ability in the absence of any stimulatory signals. The cell survival rate of PD-1^+ ^T cells was higher than those of PD-1^+++ ^cells and PD-1^++ ^cells (Figure 5D). Approximately, 15% of PD-1^+++ ^cells were Ki-67^+ ^T cells, which indicates proliferation of these T cells (Figure 5E). These results indicate that PD-1^+++ ^cells are active in proliferation in vivo but are prone to cell death and difficult to activate for proliferation in vitro.

### Roles of IL-2 and IL-7 in survival and proliferation of PD-1-expressing T cells

**Figure 6:**
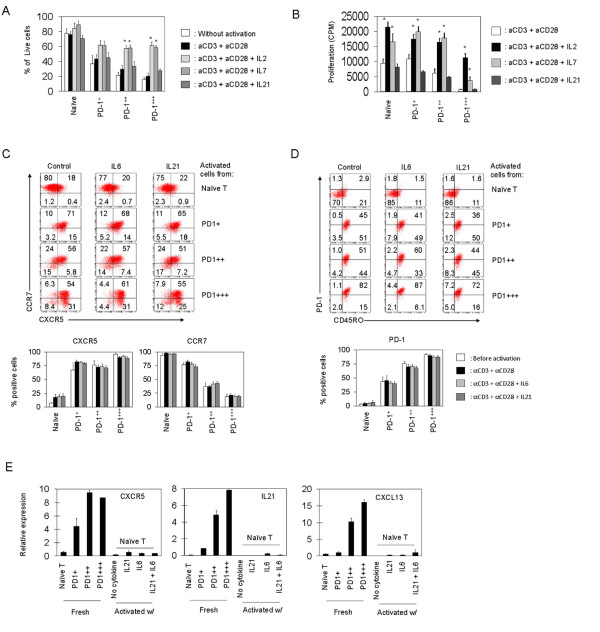
**Roles of cytokines in maintenance and induction of PD-1-expressing T cells**. Survival (A), proliferation (B), and changes in expression of CXCR5 and CCR7 (C) and PD-1 (D) by the T cell subsets were examined. (E) IL21 and IL6 fail to induce the expression of CXCR5, IL21, and CXCL13 at the mRNA level by antigen-primed naïve T cells. Relative expression levels after normalization with the expression levels of β-actin are shown in panel E. For all panels, indicated T cells were cultured for 5-6 days in the absence or presence of T cell activators with IL2, IL7 or IL21. Combined data of 3 independent experiments (A-D) or a representative real-time PCR data (E) are shown."*" and "**"indicate significant differences from naïve T cells and PD-1^++ ^cells respectively. "ND" indicates non-detectable.

Next, we examined if cytokines can regulate the survival and proliferation of PD-1-expressing T cells. Both IL2 and IL7 were able to promote the survival of PD-1^+++ ^T cells (Figure 6A). While IL2 was able to induce proliferation of PD-1^+++ ^T cells, IL7 was not effective in this activity (Figure 6B). One interesting difference between the PD-1^+++ ^cells and PD-1^++ ^cells is that IL-7 was able to readily induce the proliferation of PD-1^++ ^cells but not PD-1^+++ ^T cells, which is in line with their low CD127 expression. In contrast, IL21 had no notable effect on the survival or proliferation of the PD-1-expresing T cells. These results indicate that PD-1^+++ ^T cells can be induced for cell proliferation in an appropriate cytokine milieu.

### Lack of function for IL-21 or IL-6 in maintenance or generation of PD-1^+++ ^T cells in vitro

IL-6 and IL-21 are implicated in generation of follicular helper T cells in mice [31-33]. We examined the impact of IL-6 and IL-21 on the stability of the PD-1-expresing T cells. For this, the T cell subsets were cultured for 6-7 days in the indicated conditions, and expression of CXCR5, CCR7, and PD-1 was examined. Naïve T cells gained some expression of CXCR5 but they did not lose CCR7. PD-1^+^, PD-1^++^, and PD-1^+++ ^T cells maintained the expression of CXCR5, CCR7 and PD-1 throughout the culture period (Figure 6C and 6D). IL-6 and IL-21 had no effect on the stability of the PD-1-expresing T cells in terms of expression of PD-1, CXCR5 and CCR7.

We consider that expression of CXCR5, PD-1, CXCL13, and IL21; and loss of CCR7 are the features of mature follicular helper T cells that can effectively help B cells and are localized in the GC [30,33-37]. We examined if IL-6 and/or IL-21 has any role in generation of the PD-1-expressing CXCR5^+ ^B cell-helping T cells. We observed that these cytokines did not promote the expression of CXCR5, IL-21, or CXCL13 by antigen-primed human naïve T cells at the mRNA (Figure 6E) and protein level (not shown).

### PD-1 triggering restrains the B cell-helping ability of PD-1^+++ ^T cells in a cell-specific manner

**Figure 7:**
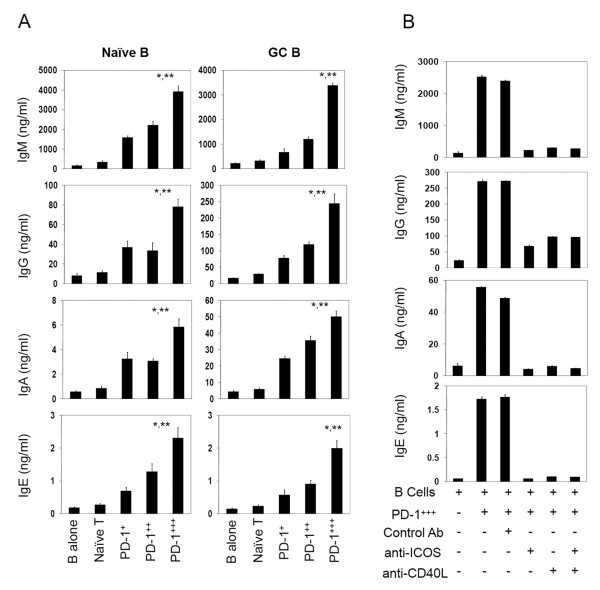
**B cell-helping activities of PD-1-expressing T cells**. (A) B cell-helping activities for naïve B cells and GC B cells. (B) Roles of ICOS and CD40L in B cell-helping activities of PD-1^+++ ^T cells. Equal numbers of highly purified T cells and B cells (naïve B cells or GC B cells) were cultured for 5 days in the presence of SEB and indicated neutralizing antibodies, and antibody production by B cells was assessed by ELISA. Combined data of 3 independent experiments are shown. "*" and "**"indicate significant differences from naïve T cells and PD-1^++ ^cells respectively.

The information that PD-1^+++ ^T cells are found in GC and express B cell-helping effector molecules such as ICOS and CD40L suggests that PD-1^+++ ^T cells are highly specialized effector T cells for helping B cells. We co-cultured the PD-1-expressing T cells and B cells and assessed their B cell-helping activity. As shown in Figure 7A, there is a striking positive correlation between the PD-1 expression and the ability to promote B cell antibody (IgG, IgA, IgE, and IgM) production. This was true for both naïve B cells and GC B cells as target cells. Neutralizing antibodies to ICOS and CD40L almost completely abolished the B cell-helping activities of PD-1^+++ ^T cells (Figure 7B).

**Figure 8:**
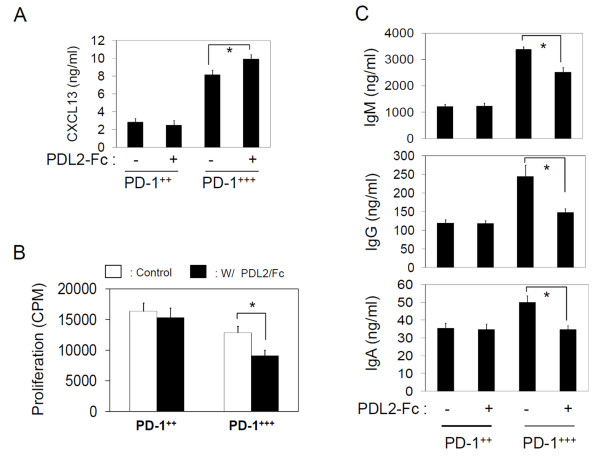
**The impact of PD-1 stimulation on the biology of PD-1-expressing T cells**. The PD-1-expressing T cells were cultured in various conditions in the presence or absence of PD-L2-Fc protein. CXCL13 production (A), cell proliferation (B), and B cell-helping activities (C) were determined. The data in panels A and B were from T cell only cultures. The cells were activated with antibodies to CD3 and CD28 in the presence of IL-2. Combined data of 3 independent experiments are shown. "*"indicates significant differences.

A question that remains to be answered regarding the function of PD-1^+++ ^T cells is about the role of PD-1 expressed by the T cells. Utilizing PD-L2-Fc fusion protein, we stimulated the PD-1 receptor of the T cells. We used a PD-L2-Fc fusion protein, but not a PD-L1 protein because PD-L1 can trigger also B7-1 in addition to PD-1 [38]. PD-1 stimulation moderately increased the CXCL13 production but decreased the proliferation and B cell-helping ability of PD-1^+++ ^T cells (Figure 8). However, the same stimulation had no effect on PD-1^+ + ^T cells. This suggests an overall negative role for the PD-1 specifically expressed by the PD-1^+++ ^GC-T cells.

## Discussion

We investigated the phenotype and function of PD-1-expressing T helper cell subsets in human tonsils. Unexpectedly, PD-1 is expressed by all memory, but not naïve, T cells in tonsils. The PD-1-expressing T cells, however, are heterogeneous in the PD-1-expression level, trafficking receptor phenotype, tissue localization, and effector function in helping B cells. PD-1^+++ ^T cells can stimulate GC B cells for generation of plasma B cells within the GC, while PD-1^dim (+/++) ^T cells are enriched with Th1 and Th17 cells, reside in the mantle zone of GC or interfollicular area, and have weaker B cell-helping activity. Interestingly, PD-1 triggering limits the activity of PD-1^+++ ^T cells in helping B cells.

Expression of several receptors such as CXCR5, CXCR4 and CCR7 is the major determining factor for localization of lymphocytes in lymphoid tissues that are divided into T and B cell areas including the interfollicular area, GC, and mantle zone. Coordinated expression of the three receptors regulates the exact microanatomical positioning of lymphocytes in mice [36,39]. This is because the chemokine ligands that bind the receptors are differentially expressed in the T cell area (CCL19 and CCL21, CCR7 ligands), the B cell area (CXCL13, the CXCR5 ligand), and certain areas of GC/interfollicular area (CXCL12, the CXCR4 ligand) [40-43]. The expression patterns of CXCL12 in mice and humans are somewhat different from each other. In mice CXCL12 is more expressed in the GC dark zone [44], while it is expressed at mRNA level by specialized reticulum cells that surround GC [45] or at the protein level in the mantle zone and interfollicular area in human tonsils as shown in this study. The chemokine receptor phenotypes of PD-1 expressing T cell subsets are in line with the expression sites of the three chemokines. PD-1^+++ ^T cells are CXCR4^++ ^CXCR5^++ ^CCR7^low ^and reside in the rim of GC adjacent to the mantle zone. PD-1^++ ^T cells are CXCR4^+ ^CXCR5^+ ^CCR7^low^, which would put them elsewhere in GC. The CXCL12 expressed in the mantle zone would attract PD-1^+++ ^T cells but these cells also highly express CXCR5 to stay within the GC close to the mantle zone. PD-1^++ ^T cells express CXCR5 but not CCR7 and stay within the GC. They express CXCR4 at a reduced level and, thus, are more scattered throughout GC instead of localized to the rim of GC. PD-1^+ ^T cells express both CXCR5 and CCR7 at a moderate level and would stay in the peri/interfollicular area outside of GC as the result of the balanced chemoattraction between CXCL13 and CCR7 ligands (CCL19 and 21).

An interesting function of human GC-T cells is to produce CXCL13 [30], which is thought to attract CXCR5^+ ^T cells, B cells, dendritic cells, and follicular dendritic cells to form and expand GC. Interestingly, the CXCL13 production ability of PD-1-expressing T cells is positively associated with their PD-1 expression level. We found that the GC-localized PD-1^+++ ^T cells are highly efficient in production of CXCL13. Other features of these PD-1-expressing GC-T cells include expression of LFA3, CD40L, ICOS, OX40, and CD10. LFA3 is important for cell-cell interaction, and thus, is likely to play a role in their interaction with other cells for cell activation and effector function [46]. ICOS, CD40L and OX40 are co-stimulatory receptors which would be involved in activating target cells (e.g. B cells) or the T cells themselves. We observed that the ICOS and CD40L signals are required for the optimal B cell-helping activity of PD-1^+++ ^T cells. The function of CD10 in GC is unclear at this time but it is likely that PD-1^+++ ^T cells would be a normal counterpart of CD10^+ ^angioimmunoblastic T-cell lymphoma in human patients [47,48].

It has been determined previously that GC-T cells are prone to cell death and do not proliferate well [49]. Some even called the cells anergic [50]. The dilemma in calling them "anergic" is that many T cells in GCs are active in cell cycling [22] and they are functionally active in producing IL21 and CXCL13 and helping B cells. These functions cannot be performed, at least by definition, by anergic T cells. Moreover, PD-1^+++ ^T cells, although as suggested before to be apoptotic and non-proliferative, can be driven to survive and proliferate in response to IL-2. IL-2 and IL-7 can rescue the T cells from cell death. Active proliferation of PD-1^+++ ^T cells in vivo is supported by expression of Ki-67 by many PD-1^+++ ^cells in tonsils. These results suggest that the PD-1^+++ ^GC-T cells are actively propagating in the GC environment in response to antigen presenting cells and cytokines. We noted also that the PD-1^+++ ^T cells were highly stable and did not lose their phenotype in expression of PD-1 and CXCR5 and in B cell-helping function upon subsequent antigen stimulation.

It is still controversial what directly regulates the generation of fully committed B cell-helping T cells, which express IL21, CXCL13, CXCR5 and PD-1 but not CCR7. ICOS, IL-21, and IL-6 are implicated in increasing the number of CXCR5-expressing T cells or B cell-helping T cells in mice [32,33,51]. As internal transcription factors, Bcl6, c-Maf, STAT3 and BATF1 are implicated in regulating the number of CXCR5^+ ^or B cell-helping T cells in vivo [51,52]. IL-12, a cytokine for Th1 cells, can increase the CXCR5 and IL21 expression by human T cells [53]. Also, IL-4 can increase the CXCR5 expression by naïve T cells in response to activation by dendritic cells [54]. We found in this study that IL6 and IL-21 lack the ability to induce the fully differentiated GC-T cells in vitro. One should note that CXCR5 induction is a spontaneous event following T cell priming in vivo [54], and is not sufficient by itself to define fully committed B cell-helping effector T cells. Also, IL21 is widely produced by different T cell types, and, thus, expression of IL21 alone would not be sufficient to define fully differentiated GC-T cells. In this regard, to date, none really established a reproducible in vitro system to induce a stable B cell-helping effector T cell lineage with a GC tropism (PD-1^+++ ^CXCR5^+ ^CCR7^-^). Our results point out that there are several subsets of CXCR5^+ ^T helper cells with distinct phenotype and cell function. "Follicular T helper cells," defined by CXCR5 expression alone, is an ambiguous term, and these T cells are actually comprised of heterogeneous T helper subsets. Additional phenotypes such as effector function, tissue localization and expression of additional antigens such as CCR7 should all be considered in characterization of the diverse B cell-helping T cells.

A key question would be "what is the function of PD-1, highly expressed by GC-T cells?" It has been established that mice deficient with PD-1 expression are prone to develop various autoimmune diseases [10,11]. Some of these autoimmune diseases are induced, in part, by autoantibodies produced by B cells, a process promoted by GC-T cells. Thus, we hypothesized that PD-1 has the function of down-regulating the T-cell-dependent B cell responses. To test this hypothesis, we stimulated the PD-1 by PD-L2-Fc protein and observed that the PD-1^+++ ^T cell-dependent antibody response was decreased. Also, observed was suppressed proliferation of PD-1^+++ ^T cells by the PD-1 triggering. However, the production capacity of CXCL13 was not altered. These results suggest that PD-1 can limit the magnitude of GC-T cell response. The degree of suppression following PD-1 triggering was moderate which suggests that PD-1 triggering would not completely shut down the GC-T cell response. Hyper-activity of PD-1^+++ ^T cells would cause chronic inflammation or autoimmune diseases. PD-1 appears to function to restrain the function of PD-1^+++ ^T cells possibly to prevent the aberrant humoral immune responses.

## Conclusions

PD-1-expressing human T helper cells are highly heterogeneous, including PD-1^high (+++)^, PD-1^medium (++)^, and PD-1^dim (+) ^cells in tonsils. Among them, PD-1^+++ ^T cells have the phenotype of the germinal center T helper cells in tissue localization, cellular phenotype and effector function. Triggering of PD-1 restrains the B cell-helping activity and proliferation of PD-1^+++ ^germinal center T cells, supporting the role of PD-1 in promoting tolerance in humoral immunity.

## Methods

### Isolation of T cells expressing PD-1 at different levels using a novel antigen density-dependent magnetic sorting (ADD-MS)

The use of human tonsils for this study has been approved by the institutional review board at Purdue. The specimens, byproducts of surgeries and obtained as pathological specimens without any associated patient information, were exempted from obtaining consent forms by the review board. Human tonsil specimens were obtained from young patients (3-10 yr) undergoing tonsillectomy to relieve obstruction of respiratory passages and improve drainage of the middle ear and had no apparent inflammation. Tonsil mononuclear cells were prepared by density gradient centrifuge on histopaque 1077 (Sigma-Aldrich, St. Louis, MO). T cells were enriched from the mononuclear cells by a sheep red blood cell (SRBC) rosetting method. CD4^+ ^T cells were isolated by the CD4^+ ^T cell isolation kit (Miltenyi Biotec Inc. Auburn, CA). CD25^+ ^(Treg-enriched) T cells were depleted with anti-CD25/magnetic beads to obtain CD4^+^CD25^- ^cells. Naïve and memory CD4^+ ^T cells were isolated by depleting CD45RO^+ ^and CD45RA^+ ^cells respectively. To isolate the T cell subsets, we developed a novel magnetic sorting method utilizing controlled bead release based on antigen expression density. The advantage of this method is to isolate cells at a relatively high speed using a magnetic sorting method utilizing commercially available magnetic beads (anti-FITC/PE beads, Miltenyi Biotec Inc). This method was more efficient than a flow cytometry method in preparing many cells (1- 5 million sorted cells) within a short time period (5 h). PD-1^+/- ^T cells were isolated by magnetic sorting from the CD4^+^CD25^-^CD45RA^- ^cells by magnetically selecting PD-1^++/+++ ^T cells. PD-1^+ ^memory T cells were further isolated from the PD-1^+/- ^T cell fraction by magnetically selecting PD-1-expressing cells. PD-1^++/+++ ^T cells were cultured for 30 min at 37°C in complete RPMI-1640 to release attached beads from PD-1^++ ^cells. PD-1^+++ ^T cells were positively selected from the cultured PD-1^+ +/+++ ^fraction. The negative fraction of this isolation was used as PD-1^++ ^T cells. All of the T cell fractions were highly pure (> 97% based on the expression of CD4 and CD45RO or CD45RA). Figure 4 shows the typical purity of PD-1-expressing T cells isolated by this method. Total CD19^+ ^B cells were isolated by depleting T cells with a SRBC-rosetting method. Naïve B cells were further isolated from the total B cells by positively selecting IgD^+ ^cells (purity > 99%), and CD19^+^CD38^+^IgD^- ^GC B cells (purity = ~95%) were isolated by depleting IgD^+ ^cells and positively selecting CD38^+ ^cells as described before [55].

### Expression of trafficking receptors and other antigens by T cells by flow cytometry or real time PCR

The T cells isolated with SRBC-rosetting were stained with antibodies to CCR7 (150503), CXCR4 (44717.111), CXCR5 (51505.111, all from R&D Systems, Minneapolis, MN), or mouse control IgG1 (BioLegend, CA). Cells were further stained with a biotinylated horse anti-mouse IgG (H+L) antibody (Vector Lab, Mountain View) for 20 min, followed by staining with APC-streptavidin (BD Biosciences) and antibodies to PD-1 (eBioJ105), CD45RO (UCHL1), and CD4 (RPA-T4). For surface or intracellular staining, T cells were stained with antibodies to CD4 (RPA-T4), CD45RO (UCHL1), PD-1 (eBioJ105), CD58 (LFA-3, 1C3), CD134 (OX40, ACT35), CD10 (HI10a), CD127 (hIL-7R-M21), CD62L (Dreg 56), CD69 (FN50), ICOS (C398.4A), CD25 (BC96), FOXP3 (259D), Ki-67 (B56), and/or CD154 (TRAP-1). The antibodies to the antigens were purchased from R&D systems, eBioscience, BioLegend, or BD Biosciences (San Jose, CA). Intracellular staining for cytokine production was performed as described previously [56]. Stained cells were acquired on a FACS Canto II. Real time PCR detection was performed on cDNA with a 7500 Sequence Detection System (Applied Biosystems, Foster City, CA) using the SYBR green Master Mix (Applied Biosystems). Primers used were: hIL-21-F (TTC TGC CAG CTC CAG AAG ATG), hIL-21-R (CAC TTC CGT GTG TTC TAG AGG), h-CXCR5-F (GCC AGA GAT TCT CTT CGC CAA), h-CXCR5-R (TGT CCA GGA AGA TGA CGA TGT G), h-CXCL13-F (TCC AAG GTG TTC TGG AGG TC), and h-CXCL13-R (TTT CTT GGA CAA CCA TTC CC).

### Immunohistochemistry

Frozen sections of tonsils were cold acetone-fixed and stained with monoclonal antibodies to human CD3 (HIT3a), CD4 (RPT-T4), PD-1 (eBioJ105), CD19 (HIB19), CD21 (BU32), CD11c (3.9), and/or IgD (IA6-2). For detection of human CXCL12, the acetone fixed sections were stained with anti-CXCL12 (Clone 79018; R&D systems) and then with biotin-conjugated horse-anti-mouse IgG (H+L) (Vector lab). The sections were further stained with FITC or PE-conjugated streptavidin (eBioscience). After blocking with 10% mouse serum, the sections were stained with an antibody to PD-1. Slides were examined with a microscope equipped with epifluorescence as described previously [49].

### Chemotaxis

Chemotaxis was performed as described previously [57]. Human CXCL12, CCL19 and CXCL13 were purchased from R&D Systems. 5 ×10^5 ^CD4^+ ^T cells in 100 μl of chemotaxis medium (RPMI with 0.5% BSA) were placed in each Transwell insert (5 μm pore, 24-well format; Corning Costar Corp., Cambridge, MA), and the Transwell inserts were placed in 24-well plates containing 600 μl of chemotaxis medium (RPMI-1640 with 0.5% BSA) with optimal concentrations of CCL19 (2000 ng/ml), CXCL12 (100 ng/ml) or CXCL13 (3000 ng/ml). Cells were allowed to migrate for 3 h in a 5% CO_2 _incubator at 37°C. After chemotaxis, the cells that migrated to the lower chambers were harvested and stained with antibodies to CD4 (RPA-T4), PD-1 (eBioJ105 (J105)), and CD45RO (UCHL1). Stained cells were acquired on a FACS Canto II, and specific percent migration after subtraction of the background migration was calculated.

### Proliferation, cell survival, differentiation and CXCL13 production

Sorted T cells were cultured in U-bottomed 96-well plates for 5-6 days in the presence of phytohemagglutinin (PHA, 5 μg/ml) or anti-CD3 (5 μg/ml, immobilized) and anti-CD28 (2 μg/ml, soluble) in the presence of hIL-2 (20 U/ml), hIL-7 (20 ng/ml), hIL6 (20 ng/ml), and/or IL21 (50 ng/ml). For assessment of proliferation, cells were further incubated with 1 μCi/well of ^3^H-thymidine for 8 hours, and ^3^H-thymidine incorporation was measured by a beta scintillation counter (Packard Top Count Microplate Scintillation Counter, Packard Instruments, Meriden, CT). For the cell survival assay, isolated T cell subsets were cultured for 5 days in RPMI/10% FBS and, then, stained with 7-Amino-actinomycin D (7-AAD; final 0.5 μg/ml) immediately before flow cytometric detection of dead (7-AAD^+ ^or FSC^low^) cells. The 5-day old culture medium was examined for CXCL13 production with an anti-hCXCL13 ELISA kit (R&D Systems).

### Assessment of B cell-helping activity

To cross-link the B cell receptors, isolated B cells were incubated for 2 h at 4°C with sepharose-conjugated rabbit Ab to human Ig μ chain and human Ig (H + L) chain (Irvine Scientific, Santa Ana, CA; mixed 1:1 at 2 μg/ml) and then washed with cold PBS. Sorted T cells and B cells (10^5 ^each) were co-cultured in each well of 96-well plates in RPMI1640 medium supplemented with 10% FBS, gentamycin, streptomycin, and penicillin in the presence of Staphylococcal enterotoxin B (SEB; 1 μg/ml, Sigma-Aldrich, St. Louis, MO). When indicated, recombinant human PD-L2-Fc chimera (10 μg/ml, R&D Systems) or control antibodies (mouse IgG1, 11711.11, R&D systems) were added at 10 μg/ml to trigger PD-1.

### Statistical analyses

Student's paired 2-tailed *t *test was used for statistical analysis when indicated. *p *values < or = 0.05 were considered significant.

## List of abbreviations

GC: germinal center; MZ: mantle zone; SEB: Staphylococcal enterotoxin B; PD-1: Programmed death-1; PD-1^+^: PD-1 ^low^; PD-1^++^: PD-1^medium^; PD-1^+++^: PD-1^high^ ; PFA: perifollicular area

## Competing interests

The authors declare that they have no competing interests.

## Authors' contributions

The authors declare that there is no competing interest. CW carried out the most of the experiments and participated in the preparation of the manuscript. PH prepared the tonsil specimens. CHK conceived of the study, and participated in its design and coordination of the research and helped to draft the manuscript. All authors read and approved the final manuscript.
